# Susceptibility Status and Resistance Mechanisms in Permethrin-Selected, Laboratory Susceptible and Field-Collected *Aedes aegypti* from Malaysia

**DOI:** 10.3390/insects9020043

**Published:** 2018-04-14

**Authors:** Rosilawati Rasli, Han Lim Lee, Nazni Wasi Ahmad, Siti Futri Farahininajua Fikri, Roziah Ali, Khairul Asuad Muhamed, Azahari Abdul Hadi, Qi-yong Liu, Feng Xia Meng

**Affiliations:** 1 Medical Entomology Unit, Infectious Disease Research Centre, Institute for Medical Research, Jalan Pahang, Kuala Lumpur 505888, Malaysia; leehl@imr.gov.my (H.L.L.); nazni@imr.gov.my (N.W.A); futri_f@yahoo.com (S.F.F.F); roziahali@gmail.com (R.A); kasuadimr@gmail.com (K.A.M.); azahari.ahadi@gmail.com (A.A.H.); 2 State Key Laboratory for Infectious Diseases Prevention and Control, National Institute for Communicable Disease Control and Prevention, Chinese Center for Disease Control and Prevention, Beijing 102206, China; liuqiyong@icdc.cn (Q.-y.L.); mengfengxia@icdc.cn (F.X.M.)

**Keywords:** *Aedes aegypti*, permethrin resistance, enzyme detoxification

## Abstract

This study is intended to provide a comprehensive characterization of the resistance mechanisms in the permethrin-selected (IMR-PSS) and laboratory susceptible (IMR-LS) *Aedes aegypti* strain from Malaysia. Both IMR-PSS and IMR-LS provide a standard model for use in assessing the pyrethroid resistance in field-collected strains collected from three dengue hotspots: the Taman Seri Bayu (TSB), the Flat Camar (FC), and the Taman Dahlia (TD). Two established methods for determining the resistance mechanisms of the pyrethroid are the quantification of detoxification enzymes via enzyme microassay and the nucleotide sequencing of the domain 2 region from segment 1 to 6 via classical polymerase chain reaction (PCR) amplification—were employed. Enzyme activities in IMR-LS served as the resistance threshold reference, providing a significant standard for comparison with IMR-PSS and other field-collected strains. The amino acids in the domain 2 region of voltage-gated sodium channel (*Vgsc*) of IMR-LS were served as the reference for detection of any changes of the knockdown resistance (*kdr*) alleles in IMR-PSS and field-collected strains. Studies clearly indicated that the IMR-LS was highly susceptible to insecticides, whilst the IMR-PSS was highly resistant to pyrethroids and conferred with two resistance mechanisms: the elevated oxidase enzyme activity and the altered target-site mutations. Mutations of V1023G alone, and the combination mutations of V1023G with S996P in IMR-PSS, as well as the in field-collected *Aedes aegypti* strain, indicate the spread of the (*kdr*) gene in *Aedes aegypti*, particularly in dengue-endemic areas in Malaysia.

## 1. Introduction

The global burden of dengue is hefty and it is estimated that 50 million infections occur annually in 100 countries worldwide with potential for further spread towards previously unaffected regions due to global warming [1]. Dengue is one of the main public health concerns in Malaysia. Malaysia experienced a massive dengue outbreak in 2015, with 120,836 cases recorded, which was a three-fold increase compared to the 43,346 cases in 2013. The increased morbidity is also accompanied by the higher fatality caused by dengue [2]. *Aedes aegypti* is the primary vector of dengue in the globe, whilst *Aedes albopictus* is a secondary vector which has spread to the western hemisphere due to international trading, rapid urbanization, and increasing human movement. *Aedes* mosquitoes are predominantly found in tropical and subtropical countries and have established themselves in urban and suburban areas [3].

In Malaysia, the *A. aegypti* has been incriminated as the primary vector of dengue. This mosquito is very closely associated with human surroundings and is predominantly found indoors rather than outdoors. It is a containers-breeder, preferentially rests indoors, and oviposit in water found in man-made containers around the dwelling [4]. Recently, several *Aedes* species were also incriminated as the vectors of the global Zika virus pandemic [5]. The association between the Zika virus and neonatal microcephaly is a growing of concern in many countries [6]. The continued absence of an effective vaccine for Zika and dengue makes vector control, such as the application of insecticides, the only means available for the prevention and control of these *Aedes*-borne diseases [7]. To date, pyrethroids are the most preferred adulticides for dengue control in many countries, including Malaysia [8]. Pyrethroids are effective insect control agents and they act by binding to pyrethroid-receptor binding sites in sodium channels resulting in hyperpolarization, which prevents the repolarization phase of an action potential, thus, disrupting the normal function of voltage-gated sodium channels (*Vgsc*) [9,10]. Unfortunately, in the event of mutations in the *Vgsc* the effectiveness of pyrethroid decreases [11]. Employing the fact that resistance develops due to the increase of detoxification enzymes and the mutation of sodium channel mainly in the domain 2 region has prompted us to develop a standard reference for evaluating the resistance profile of pyrethroid resistant *A. aegypti* from Malaysia. This present study provides information on the status of pyrethroid resistance and the distribution of the knockdown resistance (*kdr*) gene in *A. aegypti*, particularly in dengue-endemic areas in Malaysia.

## 2. Materials and Methods

### 2.1. Mosquito Samples

Three different strains of *Aedes aegypti* were used in this study: the laboratory-susceptible strain, the permethrin-selected strain, and the field-collected strain. The laboratory-susceptible and permethrin-selected strains were assigned as IMR-LS and IMR-PSS, respectively, whilst the field-collected strains from four dengue hotspots were assigned according to the name of the collection sites (Table 1).

### 2.2. Mosquito Collection and Colonization

Field mosquitoes were collected via ovitrapping [12]. The eggs obtained from ovitraps hatched into larvae and the species was identified at the L3 stage of the larvae under a compound microscope (Nikon Eclipse E200, Tokyo, Japan) using established taxonomy keys [13]. They were then reared into adulthood. The field colony of the *A. aegypti* was maintained until the F2 generation and used for tests to determine their insecticide susceptibility level.

The colony of IMR-LS originated from the Selangor state, had been bred continuously for 1066 generations; the IMR-PSS originated from S15, Bandar Baru Bangi, which was collected from one of the 12 dengue-endemic localities where *A. aegypti* samples were collected and screened by Rosilawati et al. (2017). The IMR-PSS has been continuously selected against permethrin at a lethal concentration of 50% (LC_50_) for 24-h exposure at every successive generation.

Each of the colonies from the field-collected mosquitoes, IMR-PSS, and IMR-LS were maintained in separate rooms in the insectarium of the Medical Entomology Unit, the Institute for Medical Research, Kuala Lumpur 27 ± 2 °C and 75 ± 5% relative humidity. The susceptibility status of all the strains was evaluated using World Health Organization (WHO) guidelines [14].

The colonization of *A. aegypti* was based on the Standard Operating Procedure of Colonization of the *Aedes aegypti* in Medical Entomology, IMR. Briefly, the first to second instar larvae were fed with ox liver powder twice a day and later replaced with half cooked liver pieces once they reached the third instar. Later, the fourth instar larvae, which developed into pupae, were placed into a bowl consisting of clean tap-water and transferred into a cage (30 cm height × 30 cm width) for adult emergence. The adult mosquitoes were provided ad libitum with a 10% sugar solution soaked in cotton. The female mosquitoes were allowed to feed on the blood of mice on the third post-emergence day. Fully engorged females were provided with a 3-day rest period to allow for the complete development of fertile eggs. Following that, a bowl containing a filter paper (Whatman No. 1) wetted with 25 mL of tap-water was placed in the cage containing the gravid adult females. Oviposition took approximately 2–3 days and eggs were observed on the surface of the wet filter paper mostly along the water edge. The eggs were dried at 27 ± 2 °C for approximately 2–3 days before being submerged again in water for hatching.

### 2.3. Selection Pressure to Permethrin

Each generation of IMR-PSS was selected with permethrin via exposing 1000 third instar larvae against permethrin at LC_50_ for 24-h and the larvae that survived were reared and colonized for successive generations. For IMR-PSS, the LC_50_ of permethrin was determined prior to the selection pressure for every successive generation of IMR-PSS. For IMR-LS, no selection pressure was performed and the LC_50_ of each IMR-LS was determined via larval bioassay protocol.

### 2.4. Larval Bioassay

The larval bioassay was conducted according to the standard procedures described by World Health Organization with a slight modification [14]. Initially, the stock solution of permethrin at 50 mg/mL was prepared by dissolving 51.49 mg of technical grade permethrin (Sumitomo Chemical Ltd., London, UK) in 10 mL ethanol.

To establish a series of 5 baseline concentrations, 20 sets of larval bioassays of IMR-PSS larvae were set up against 20 respective concentrations of permethrin ranging from 0.001 mg/L to 1.0 mg/L, while 10 sets of larval bioassays of IMR-LS larvae were set against 10 respective concentrations of permethrin ranging from 0.001 mg/L to 0.05 mg/L. The appropriated volume of permethrin was pipetted into a paper cup, topped up with 250 mL of seasoned water, and stirred vigorously for 30 s with a glass rod. Then, 25 third instar larvae were added into a paper cup. Each concentration of permethrin was run in three replicates with a slight modification. For the control, 1 mL of ethanol was added into the paper cup and topped up with 250 mL of seasoned water. They were also run in three replicates. The temperature was maintained at 27 ± 2 °C throughout the experiments. The mortality was observed at 24-h post exposure. Subsequently, 5 concentrations yielding a larval mortality between 5% to 100% post 24-h exposure were used to determine the lethal concentration of LC_50_ via Probit analysis.

### 2.5. Adult Bioassay Test

This test was conducted by following the WHO protocol [14]. Female adult mosquitoes, sugar-fed and aged 3–5 days from the field-collected *A. aegypti* strain at the F2 generation and the IMR-PSS strain at the F1 and F7 generations were exposed to diagnostic concentrations of pyrethroids (0.03% deltamethrin, 0.25% permethrin, 0.15% cyfluthrin, and 0.03% lambda-cyhalothrin) and to organophosphate (0.8% malathion and 1% fenitrothion) impregnated paper at the recommended exposure time. The IMR-LS strain was served as the control strain. Four replicates of treated mosquitos and 3 control tubes were prepared. The maximum recommended number of 25 mosquitoes per replicate was used. The mosquito knockdown was observed at regular intervals and counted during the exposure time. After one hour of exposure, the mosquitoes were transferred into a holding tube for a 24-h recovery period. Cotton wool soaked in a 10% sugar water solution was placed on top of the holding tubes, which were in an upright position. The number of dead mosquitoes was counted and recorded at the 24-h post exposure mark. The mortalities of all treated tubes were corrected using Abbot’s formula if more than a 5% mortality rate was recorded in control tubes [15]. A similar protocol described above was conducted for assessing the susceptibility status of IMR-PSS at every generation.

### 2.6. Enzyme Microassays

Three enzyme assays, namely mixed function oxidase (MFO), esterase, and insensitive acetylcholinesterase (AChE) were conducted to determine the enzymes activities in the IMR-LS and the IMR-PSS, while only the MFO assay was conducted for the field-collected strain. The same homogenate of the individual mosquito was used to assess all three enzymes activities.

#### 2.6.1. Preparation of the Mosquito Homogenate for Enzyme Microassays

A potassium phosphate buffer (PBS) was prepared by dissolving 79.27 mg of potassium phosphate in 100 mL of distilled water. The buffer solution was adjusted to a pH of 7.2. Then, 0.2 mL of the PBS buffer was pipetted into a 1.5 mL Eppendorf tube containing an individual mosquito, followed by the grinding of the mosquito using a pestle mixer (Thermo Scientific, Waltham, MA, USA). Finally, added in 0.4 mL of PBS buffer, making to a final volume of 0.6ml of mosquito homogenate. Only sugar-fed female mosquitoes, aged 3–5 days were used for the enzyme microassay. All steps pertaining to the preparation of the mosquito homogenate was performed on ice in order to avoid enzyme degradation. The homogenate was centrifuged at 15,000 rpm at 4 °C for 30 min. The pellet was discarded and the supernatant was used as the enzyme source for enzyme microassay.

#### 2.6.2. MFO Assay

The protocol of MFO assay described by Brogdon [16] and Nazni et al. [17] with slight modifications was employed. Firstly, a substrate solution of 2 mM of 3,3’,5,5’,-tetramethylbenzidine (TMBZ) was prepared by dissolving 5 mg of TMBZ in 2.5 mL of absolute methanol and mixing it with 7.5 mL of 0.25 M sodium acetate buffer (pH 5.0). Four replicates of 100 μL individual mosquito homogenate were filled vertically into a 96-well microplate. A total of 200 μL of substrate solution was added into each well containing the homogenate and mixed thoroughly. The reaction was allowed to continue under room temperature for 10 min and then stopped by adding 25 μL of 3% H_2_O_2_. The optical density (OD) was determined at 630 nm using an immunoassay reader (Thermo Scientific, Waltham, MA, USA).

#### 2.6.3. Non-Specific Esterase Assay

Esterase assay was performed by following the protocol described by Brogdon et al. [18] and Lee [19]. A total of 50 μL of alpha-naphthyl acetate was added into each well containing 50 μL of mosquito homogenate and incubated at room temperature for 15 min. This substrate was prepared by weighing 14 mg of alpha-naphthyl acetate and completely dissolving it in 5 mL of acetone before adding it into 20 mL of phosphate buffer (0.5 M). Thereafter, 50 μL of Fast Blue B stain was added for color development. The absorbance of the enzymatic reaction was measured after 10 min at 570 nm.

#### 2.6.4. AChE Assay

The modification of Ellman’s method described by brogdon et al. was followed [18,20]. The enzyme activities of AChE in 25 µL of mosquito homogenate were initiated by the addition of a substrate solution of acetylthiocholine iodide (ACTHI). The substrate solution was prepared freshly by dissolving 37.5 mg of ACTHI in 5 mL of acetone and topped up with a phosphate buffer to a final volume of 50 mL. Four replicates of an individual mosquito homogenate were used; whereby 2 replicates were allowed to progress normally, while the reaction in the remaining 2 replicates were inhibited by 2 mg/L of Propoxur. A faint colorless solution would indicate the presence of sensitive AChE in the replicates inhibited by Propoxur, while an intense yellowish solution would indicate the AChE insensitivity since the enzyme would not be inhibited by Propoxur. The OD value of all reactions was measured at 410 nm after 30 min of incubation at room temperature.

#### 2.6.5. The Determination of the Protein Content in Mosquitos

Only 20 µL of the homogenate of mosquitos was used to determine the protein content in each homogenate. A similar arrangement of mosquito homogenates that were filled in 96 well plates as in the enzyme microassay was followed. This was to ensure the accurate measurement of each individual protein that corresponds to each individual mosquito used. Eighty µL of PBS was transferred into each well containing the homogenate, followed by adding 160 µL of Bradford assay and incubating it for 5 min. Later on, the protein absorbance was read at a wavelength of 560 nm. The protein content (µg/µL) was determined by referring to the standard curve of absorbance for a known concentration of bovine serum albumin BSA [21].

#### 2.6.6. Determination of Enzyme Activity

The mean optical density (OD) of the four replicates derived from an individual mosquito was determined. The cytochrome C and esterase activity of each mosquito was determined using a standard equation curve developed from the absorbance of a known serial concentration of cytochrome C and alpha-naphthol, for the MFO and esterase microassay, respectively. The cytochrome activity was expressed in nmol cytochrome C/min/mg of protein, while the esterase activity was expressed in nmol alpha-naphthol/min/mg of protein. For AChE activity, the residual AChE activity in an individual mosquito was determined by dividing the value of the mean OD of the well with Propoxur by the mean OD of the well without Propoxur (given as a percentage).

The enzyme data of each strain were pooled and the frequency of distribution was computed. The upper value of the enzyme activity of MFO and esterase, and the maximum frequency of the IMR-LS population that inhibited AChE were set as the cut off points for the determination of the resistance threshold. The classification of all three enzymatic activities of IMR-LS and IMR-PSS was constructed. High (+++), moderate (++), and low (+) scores were used to classify the MFO, esterase, and AChE activities based on the heterogeneity of the resistance distribution.

### 2.7. The Partial Amplification of Domain 2, Segment 1–6, Voltage-Gated Sodium Channel in Aedes aegypti

The mutations in the voltage-gated sodium channel in the IMR laboratory susceptible, permethrin-selected, and field-collected *A. aegypti* strains were examined using the primers IIS1-6_F and IIS 6_R [22]. The total RNA was extracted from the individual adult mosquito using the RNeasy Mini Kit (Qiagen, Hilden, Germany) by following the manufacturer’s instruction. The RNA was aliquoted into several tubes and was stored in a freezer at −80 °C prior to polymerase chain reaction PCR amplification using the OneStep RT-PCR kit (Qiagen, Hilden, Germany, Cat. No. 210210). The kit allows for both reactions of RNA conversion to cDNA and PCR amplification to take place in the same tube during the PCR reaction. After reverse transcription at 50 °C for 30 min, during which RNA was transcribed to cDNA, the reactions were then heated to 95 °C for 15 min in order to activate the HotStarTaq DNA while inactivating the reverse transcriptase. PCR cycling was continued and the DNA template was denatured at 94 °C for 1 min, followed by the annealing of primers at 59 °C for 1 min, and the DNA extension at 72 °C for 1 min. The PCR cycle was repeated 35 times and terminated with the final extension at 72 °C for 10 min. The amplified fragment was electrophoresed on 1.0% agarose gel, stained with GelStar™ Nucleic Acid Gel Stain (Lonza, New Jersey, NJ, USA), and viewed later under a UV light. The PCR fragment was then extracted and purified using the QIAquick PCR purification kit (Qiagen, Hilden, Germany) and the product was sent for sequencing.

### 2.8. The Analyses of cDNA and Amino Acid Sequences of the Domain 2, Segment 1–6, Aedes aegypti Sodium Channel

The amplified cDNA region for both reverse and forward sequences were assembled using CodonCode Aligner (Version 7.1, CodonCode Corporation, CenterVille, OH, USA). The cDNA sequence was aligned using the Clustal Omega web-based tool (http://www.ebi.ac.uk/Tools/msa/clustalo/). The comparison of amino acids between *A. aegypti* samples was conducted and the mutant samples were determined.

## 3. Results

### 3.1. Resistance Profiles of Permethrin-Selected (IMR-PSS), Laboratory-Susceptible (IMR-LS), and Field-Collected Aedes aegypti Strains

#### 3.1.1. WHO Larval Bioassay

The LC_50_ of the IMR-PSS third instar larvae selected with permethrin from the F1 to F7 generations and the IMR-LS larvae from the F1061 to F1066 generations were summarized in Table 2 and Table 3, respectively. IMR-PSS was derived from the wild-caught resistant population (IMR-PSS F0). The continuously applied selection pressure to the IMR-PSS F0 strain against permethrin from the early generations of F0 to F7 showed an increment of the LC_50_ value by a mean of 1.16 folds as the generation increased (Table 2). The highest LC_50_ value recorded among all other generations was at the F7 generation, with 1.020 mg/L of permethrin (Table 2). On the contrary, there was no significant difference in the LC_50_ value between the 6 LS generations due to the absence of selection pressure on this strain (Table 3).

#### 3.1.2. WHO Adult Bioassay

**Figure 1:**
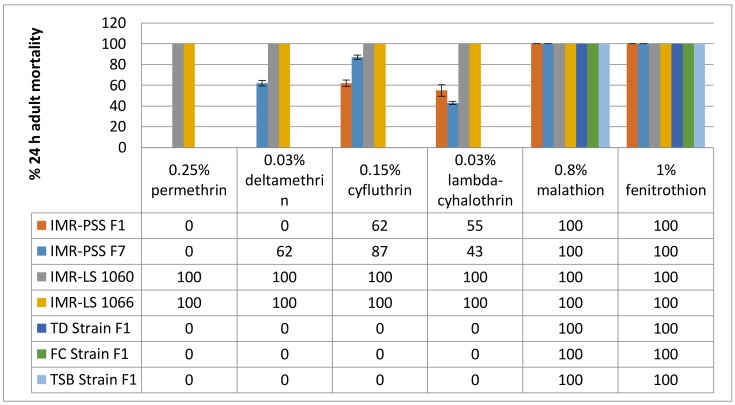
The adult susceptibility of all three *Aedes aegypti* strains to WHO diagnostic concentrations of pyrethroids and organophosphates.

The adult susceptibility status of IMR-PSS (the F1 and F7 generations), IMR-LS (F1060 and F1066 generations), and field-collected strains, to diagnostic concentrations of pyrethroids and organophosphates is shown in Figure 1. The results clearly indicated that IMR-PSS was highly resistant to pyrethroids as it was recorded with less than 90% adult mortality against pyrethroid insecticides, while the complete mortality was observed when this strain was exposed to malathion and fenitrothion, indicating a high level of susceptibility to these two organophosphorus insecticides (Figure 1).

IMR-PSS at the F1 and F7 generation displayed different pyrethroids resistance patterns to deltamethrin and cyfluthrin. There were 62% and 25% increments of adult mortality in the IMR-PSS F7 generation against deltamethrin and cyfluthrin, respectively. On the other hand, there was a slight reduction of 12% of the adult mortality in the IMR-PSS F7 generation against lambda-cyhalothrin (Figure 1). The variable pyrethroids resistance pattern in IMR-PSS at the F7 generation indicates that the constant exposure of IMR-PSS to permethrin for 7 generations imposed a strong resistance to permethrin and show a weak impact on the resistance development against cyno-pyrethroids.

For field-collected *A. aegypti*, all three strains (TD, FC, and TSB) were highly resistant to pyrethroids since a complete absence of adult mortality were observed. On the other hand, they were highly susceptible to organophosphate since a complete adult mortality was observed (Figure 1).

#### 3.1.3. Knockdown Profiling

The time-to-knockdown (KT_50_) of IMR-PSS exposed to pyrethroids were significantly higher than IMR-LS. The results indicated that IMR-PSS was highly resistant to all pyrethroids, especially to permethrin (with a low knockdown rate), followed by lambda-cyhalothrin, deltamethrin, and cyfluthrin, with resistance ratios of 5.3, 4.7, and 4.1, respectively (Table 4).

### 3.2. Resistance Mechanisms of Permethrin-Selected (IMR-PSS), Laboratory-Susceptible (IMR-LS), and Field-Collected Aedes aegypti Strains

#### 3.2.1. Enzyme Bioassay Test of IMR-PSS and IMR-LS

**Figure 2:**
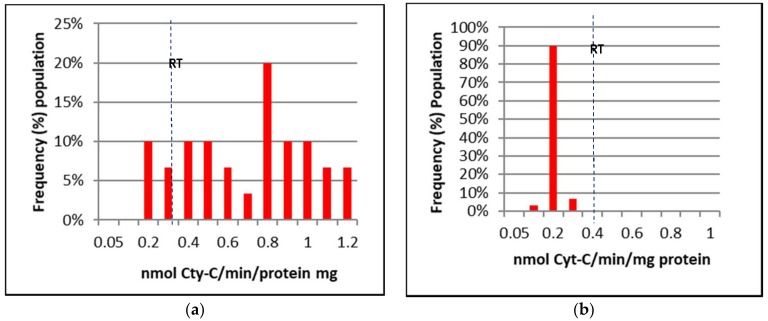
The resistance thresholds (RT) of mixed-function oxidase (MFO) enzyme activities in adult *Aedes aegypti* (**a**) MFO activities of IMR-PSS (F7 generation); (**b**) IMR-LS adult *Aedes aegypti* (F1066 generation).

**Figure 3:**
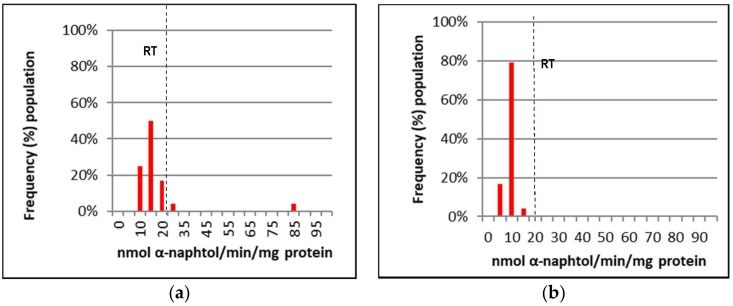
The resistance thresholds (RT) of non-specific esterase activities in adult *Aedes aegypti*; (**a**) non-specific esterase activities of IMR-PSS (F7 generation); (**b**) non-specific esterase activities of IMR-LS (F1066: generation).

**Figure 4:**
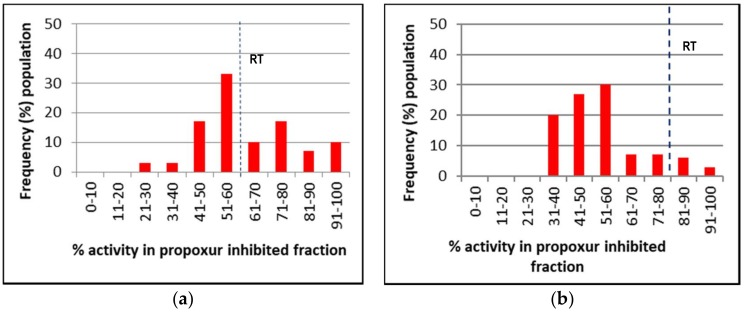
The cut-off point value of IMR-LS was used to define homozygous susceptible (SS), heterozygous resistant (RS), and homozygous resistant (RR); SS indicated a >70% frequency population having <60% activity of the Propoxur inhibited fraction, RS indicated a ~50% frequency population having <60% activity of the Propoxur inhibited fraction; RR indicated a >70% frequency population having >60% activity of the Propoxur inhibited fraction. (**a**) The altered acetylcholinesterase (AChE) activities of IMR-PSS; (**b**) the altered acetylcholinesterase (AChE) activities of IMR-LS.

Figure 2, Figure 3 and Figure 4 represent the mixed-function oxidases (MFO), esterase, and altered acetylcholinesterase (AChE) enzymatic activities of IMR-PSS and IMR-LS that were individually assayed.

The frequency of IMR-LS and IMR-PSS with cytochrome C activity (nmol cytochrome C/min/mg of protein) >0.7, 0.4 to 0.7, and 0.1 to 0.3 were pooled and grouped into high, moderate, and low, respectively (Figure 2 and Table 5), while esterase activity (nmol α-naphtol/min/protein mg) >70, 20 to 69, and <20 were similarly grouped into high, moderate and low, respectively (Figure 3 and Table 5). For AChE activities, the inhibition of 60% of AChE by Propoxur activities as observed in IMR-LS was set as the maximal value (Figure 4). The ranges of the high, moderate, and low groups of AChE activities was >81%, and 61% to 80%, and <60% respectively (Table 5).

Using an enzyme data-classifications scheme (Table 5), the results indicated that IMR-LS was considered a homozygous susceptible population which displayed substantially low MFO and esterase activity with 77% of the population showing 60% of AChE inhibited by Propoxur (Table 5). The high MFO activity of the IMR-PSS strain was correlated with the high level of cytochrome C activity, in which 54% of the population possessed more than 0.07 nmol of cytrochrome C/min/mg of protein (Figure 2 and Table 5). The IMR-PSS strain demonstrated low esterase activity without a significant difference compared to the IMR-LS strain (Figure 3 and Table 5). IMR-PSS also displayed a heterozygous population with 56% of the population having functional AChE (Figure 4 and Table 5).

Screening of the MFO enzyme activities in the field-collected strains by using the classification of MFO enzymes activity in IMR-PSS and IMR-LS (Table 5) indicated that 66% of the total FC strain (*n* = 30) showed high MFO activity. On the contrary, 79% of the total TD strain (*n* = 30) and 67% of the total TSB strain (*n* = 30) had low MFO activity (Table 5).

#### 3.2.2. Partial Sequencing of the IMR-PSS and IMR-LS *Aedes Aegypti* Voltage-Gated Sodium Channel (Vgsc)

**Figure 5:**
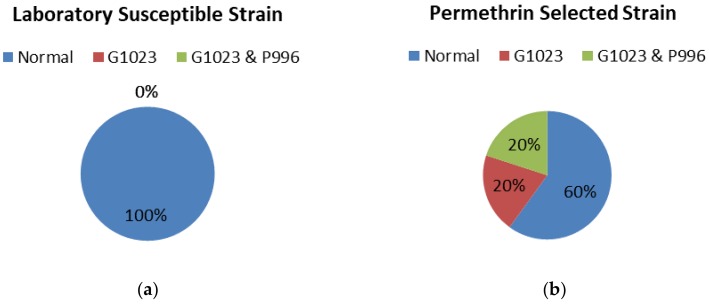
The frequency of knockdown resistance *kdr* mutations within the Domain 2 (S1–S6) Sodium Channel in IMR-PSS *Aedes aegypti* compared to IMR-LS *Aedes aegypti*. (**a**) IMR-LS without substitution mutations; (**b**) IMR-PSS with mutations of V1023G alone and combination mutations of V1023G and S996P.

The characterization of the amino acids of the amplified sequences of the domain II region (S1–S6) of the sodium channel for IMR-PSS and IMR-LS using an established primer (Yonala et al.) are shown in Figure 5.

**Figure 6:**
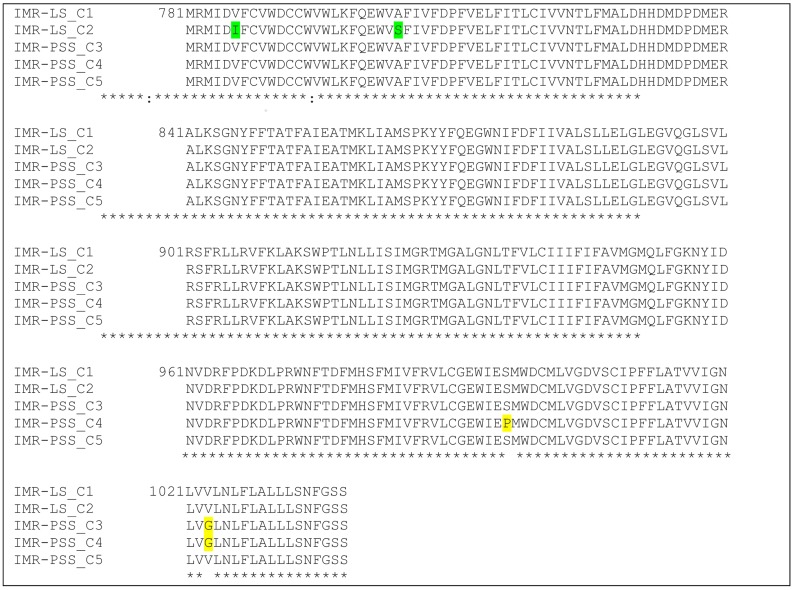
The amino acid alignment of the partially amplified domain 2, segment 1–6 of the sodium channel.

A total of 258 amino acids comprising of 812 nucleotides in both the IMR-LS strain (*n* = 15) and the IMR-PSS strain (*n* = 15) were amplified and sequenced. These nucleotide sequences are reported in the GenBank (GenBank Accession No.: MF346321-22). The amplified sequences of IMR-PSS and IMR-LS were aligned and compared with the reference sequences, namely the PMD-R (GenBank Accession No.: EU259808) strain and the China susceptible strain (GenBank Accession No.: AAT69681). Two isolates (IMR-LS_C1 and IMR-LS_C2) from IMR-LS are denoted in Figure 6. A total of 9 out of 15 amplified IMR-LS (IMR-LS_C2) sequences showed an Isoleucine (I) instead of a Valine (V) at codon 786 and a Serine (S) rather than an Alanine (A) at codon 804, whilst the rest showed identical sequences (IMR-LS_C1) when compared with the reference strain from the gene bank (Figure 6). The variation in the IMR-LS was not considered to be correlated with insecticide resistance because this strain had been established in the IMR insectarium for more than 1000 generations and confirmed to be susceptible to all insecticides tested (Figure 1 and Table 3).

For the IMR-PSS strain, it seems that IMR-PSS denotes three isolates: IMR-PSS C3, C4, and C5, respectively, which were derived from the same generation (Figure 6). Twenty percent of the IMR-PSS_C3 (GenBank Accession No.: MF346323) were identified as having the G1023 mutation, while another 20% of the IMR-PSS_C4 (GenBank Accession No.: MF346324) harbored combination mutations at G1023 and S996P; the remaining 60% of IMR-PSS_C5 (GenBank Accession No.: MF346325) showed an identical sequence as in IMR-LS (Figure 5).

**Figure 7:**
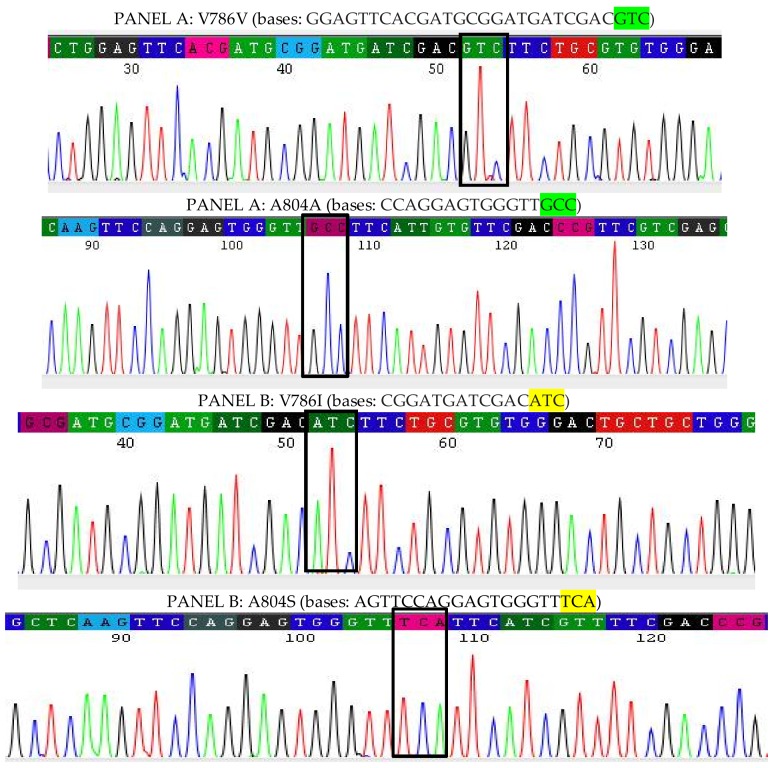
The nucleotides located in the highlighted black boxes in panel A and B belong to LS_C1 and LC_C2, respectively. The different nucleotides are shown in the highlighted boxes. IMR-LS_C2, as described in panel B, reveals a difference of amino acids: the substitution of guanine (G) to adenine (A) at the first base of codon 786, encoding valine to isoleucine; as well as the substitution of guanine (G) to thymine (T) at the first base and the substitution of cytosine (C) to adenine (A) at the second base of codon 804, encoding Alanine (GCC) to Serine (TCA).

**Figure 8:**
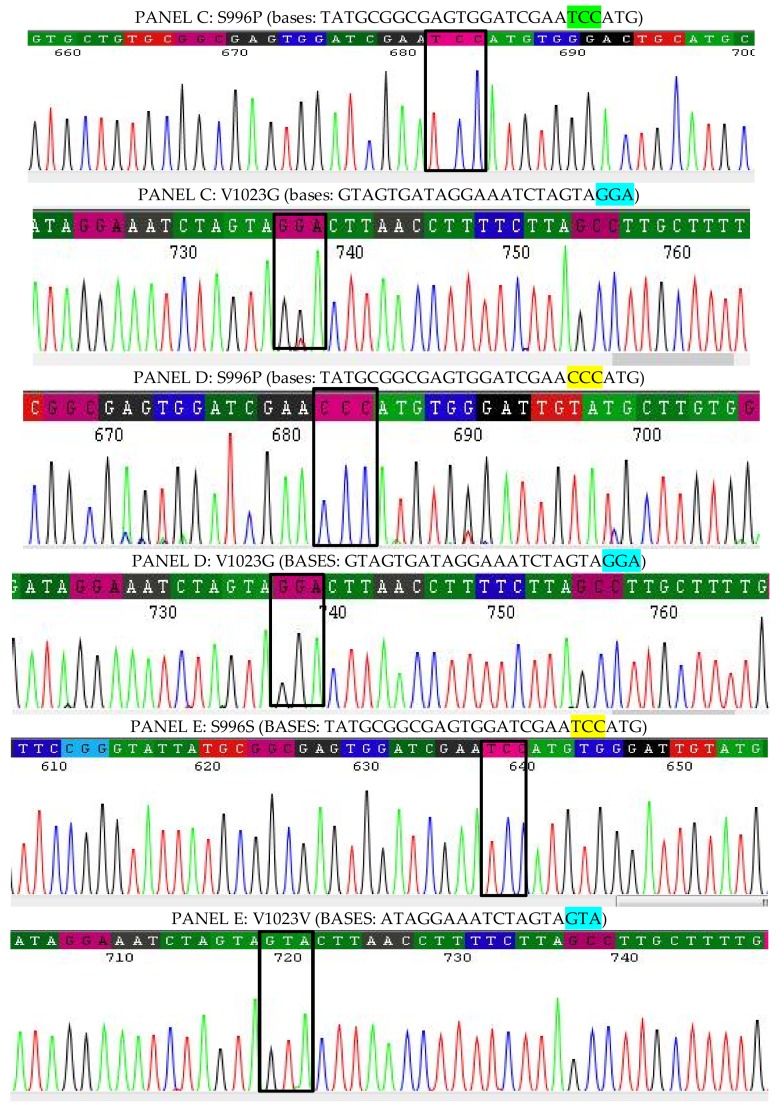
The nucleotides in panels C, D, and E belong to IMR-PSS_C5, C4, and C3, respectively. The different nucleotides are shown in the highlighted black boxes. IMR-PSS_C5, as described in panel C, revealed a single mutation of G1023 where the substitution of thymine (T) to guanine (G) at the second base of codon 1023 occurred, which is supposed to encode valine (V) instead of glycine (G). The IMR-PSS_C4 (panel D) also showed a similar mutation of G1023 with the additional substitution of thymine (T) to cytosine (C) at the first base of codon 996, giving ‘CCC’ which encodes for Proline (P) rather than Serine (S).

Further information on the electropherograms of the IMR-LS and IMR-PSS clones are displayed in Figure 7 and Figure 8.

#### 3.2.3. The Partial Sequencing of Field-Collected Aedes Aegypti Voltage-Gated Sodium Channel (Vgsc)

**Figure 9:**
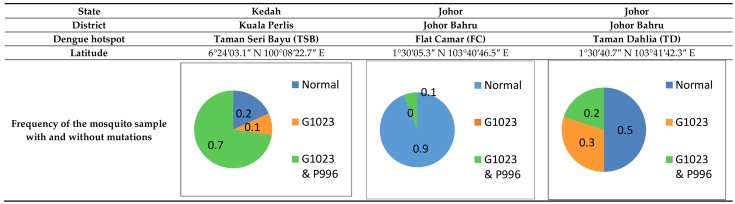
The frequency of the field-collected *Aedes aegypti* strain identified with amino acids substitution in the domain 2 (S1–S6) sodium channel.

Comparing the total amino acids of each of the 15 amplified sequences of the domain II region (S1–S6) of the sodium channel of field-collected strains with the IMR-LS and IMR-PSS strains indicated that there was an absence of other mutations in the domain 2 region of the field-collected strain except for V1023G and/or the combination mutation of S996P. The combination mutations of V1023G and S996P were found in 70% of those from TSB (*n* = 15), followed by 20% of those collected from TD (*n* = 15) and 10% of those from FC (*n* = 15). Whilst a mutation of V1023G alone was found in 10% of the TSB strain (*n* = 15) and in 30% of the TD strain (*n* = 15), respectively (Figure 9).

## 4. Discussion

Insecticides are still the forerunners in dengue and Zika control programs. The increasing trend of *Aedes*-borne viral diseases has led to the profound and widespread usage of insecticide against the mosquito vectors. Although insecticides are critical components of vector control, the unplanned and frequent use and misuse of insecticides have led to the development of a resistance against insecticides in mosquitoes, particularly in the *Aedes* vectors. Previous studies of insecticide resistance in dengue and malaria vectors have demonstrated a widespread resistance [23,24,25,26,27,28,29,30,31].

Generally, there are four major groups of insecticides, namely organophosphate (OP), organochloride (OC), carbamates (C), and pyrethroids (PY). The annual global survey on the usage of insecticides for vector control from 1995 to 2000 showed that organochlorine showed a recorded use of 5436 tons, followed by organophosphate (4000 tones), pyrethroids (120 tones), and carbamates (14 tones) [8]. Insecticides have been used in various modes of application, particularly OC and C, which are often used for residual spraying—mainly indoors. OP was applied for residual spraying, larvicide, and space spraying; whilst, PY was used for residual spraying, space spraying, and for treating bed-nets [8]. According to the WHO, only a limited number of insecticides are available for adult mosquito control. Currently, there is an absence of new adulticides for mosquito control that have been approved by the WHO in the last 15 years [8].

Many researchers have carried out research on insecticide resistance focused on the determination of the resistance status and elucidation of resistance mechanisms (i) the detection of resistance enzymes of non-specific esterase, mixed-function oxidases, insensitive acetylcholinesterase, and glutathione s-transferase, and (ii) non-enzyme based resistance mechanisms such as knockdown resistance (*kdr*) where molecular sequencing has been used successfully to characterize pyrethroid resistance [17,25,32,33,34,35].

First, the present study revealed that IMR-LS is highly susceptible to all class of insecticides while IMR-PSS is highly resistant to pyrethroids and susceptible to organophosphates. IMR-PSS exhibited an increased level of resistance to permethrin if continuously exposed to permethrin from one generation to the next. Secondly, IMR-PSS was conferred with two modes of resistance mechanisms which were high oxidase enzyme activities and the occurrence of *kdr* genes. In 2016, we conducted a nationwide monitoring of 12 dengue-endemic localities across 5 states in Peninsular Malaysia and determined that 75% of *A. aegypti* strains were highly resistant to permethrin [23]. The origin of IMR-PSS was from S15, Bandar Baru Bangi, which was collected from one of the 12 dengue-endemic localities where *A. aegypti* samples were collected and screened by Rosilawati et al. (2017). This strain is currently maintained in the Insectarium of Medical Entomology Unit. Besides using the laboratory strain as a control, it is appropriate that IMR-PSS should be included as a positive control for monitoring pyrethroid resistance in other *A. aegypti* field strains. Thirdly, field-collected *A. aegypti* from 3 dengue-endemic areas were resistant to pyrethroids and susceptible to organophosphates. The elevated mixed-function oxidase activities and genetic changes of the pyrethroid-receptor binding site were closely related to pyrethroid resistance, and both mechanisms provided protection to the insects against pyrethroids [17,32]. It was worth noting that the TD and TSB strains were resistant to pyrethroids due to *kdr* mutations, where two patterns of mutations that were similar to IMR-PSS had been circulated in both the TD and TSB strains. The FC strain was resistant to pyrethroids due to the high activity of the MFO enzyme despite the low frequency (10%) of the amplified FC strain that harbored the combination mutation of V1023G and S996P. Based on our findings, we believed that >20% of total amplified mosquito samples from the same population and generation identified with either V1023G alone or combination mutations of V1023G and. S996P could provide conclusive evidence that these *kdr* alleles could significantly contribute to pyrethroid resistance and sufficiently provide protective effects against pyrethroid.

The mutation of V1023G is not a newly reported allele in Southeast Asia, as it has been previously reported in Indonesia [31]. It was noted that V1023G plays a major role in the reduction of the sensitivity in the *Vgsc* to permethrin [31,34]. Findings from this present study clearly demonstrated that this newly detected V1023G in *A. aegypti* from Malaysia is a cause for concern, especially its impact on the effectiveness of vector control programs. Apparently, this mutation has been widely distributed across the Malaysian Peninsular. To the best of knowledge, there is a paucity of information on *kdr* studies, especially on *A. aegypti* in Malaysia. The first reported study on *kdr* genes was on the *A. aegypti* from Penang Island done by Intan et al. (2015). They identified F1534C and V1016G mutations by employing an improved pyrosequencing approach [26]. In this present study, we detected the presence of V1023G alone and V1023G combined with S996P in *A. aegypti* samples that were individually amplified using the classical PCR method.

Many studies on the *kdr* gene in mosquitoes were conducted globally. The knockdown resistance is responsible for the reduction in sensitivity of the insect nervous system to pyrethroids. The functional effects of the *kdr* gene were first described in *Musca domestica* [36,37], and later on, it was discovered in a product of the para gene in *Drosophila melanogaster* [38]. Until today, most of the *kdr* research employed both model systems (house fly and fruit fly) to determine the mutation in the sodium channel of many insects including cockroaches, mosquitoes, and agricultural pests [39,40].

There were several *kdr* mutations that have been reported in Southeast Asia, particularly in the domain 2 region of the sodium channel in *A. Aegypti*. Recent scientific findings showed that S989P, V1023G, V1016G, I110M, and F1269C were functionally proven to be associated with pyrethroid resistance [27,29,30,35,41,42]. V1016G is the most common mutation identified in selected *A. aegypti* strains originated from Yangon City, Myanmar [30], Singapore [41], Southern China [42], and throughout Thailand [29]. As for the Malaysian *A. aegypti* population, we determined that most of our strains collected from the dengue-endemic area revealed the V1023G mutation with an additional mutation of S996P instead of V1016G.

The V1023G mutation is believed to reduce the *Vgsc* sensitivity to both deltamethrin (type II) and permethrin (type I) [31,43]. S996P commonly appears together with V1023G [31]. According to Hirata et al. (2014), there is no proven effect of S996P alone on the *Vgsc* in relation to pyrethroid resistance, however, they have a slight synergistic effect when it occurs in conjunction with V1023G and increases the resistance to deltamethrin (Type II), but not to permethrin (Type I). However, a similar finding was not observed in our results because we found that *A. aegypti* from the TSB strain, which showed the highest frequency of the combination mutations of V1023G and S996P, showed little impact on the adult mortality to both permethrin (0%) and deltamethrin (0%) (Figure 1 and Figure 9).

The detection of the *kdr* gene in the dengue vector is a warning sign for the effective use of pyrethroids in the dengue control programs. Therefore, it is crucially important to screen the resistance status of the dengue vector as well as to elucidate their resistance mechanisms before this invaluable insecticide tool is rendered ineffective in killing the adult *A. aegypti.* Furthermore, monitoring pyrethroid resistance mechanisms will further aid in making judicious decisions on insecticide use, particularly in a country like Malaysia, where pyrethroid resistance of the dengue vector is not confined to certain dengue-endemic areas [23].

The findings that arose in this study displayed a standard model on assessing enzymatic activities in *A. aegypti*, as well as to determine any amino acid changes in the region 2 sodium channel using the classical PCR method. Insecticides are not and should not be the sole control agents in managing dengue vectors due to the eventual development of resistance and environmental contamination. Insecticide usage should be an integral component of new vector tools such as *Wolbachia* infected mosquitos, sterile insect technique (SIT), genetically modified mosquitos, and so forth to ensure maximal and favorable outcomes in the control of the dreadful *Aedes* borne diseases such as dengue, chikungunya, and Zika [44].

## 5. Conclusions

In conclusion, this study clearly demonstrates that the *kdr* gene and the detoxification of the oxidase enzyme play a major role in the development of a pyrethroid resistance in *A. aegypti*. Rotational planning of insecticide use by substituting pyrethroids with organophosphates is highly recommended in localities where *A. aegypti* is reportedly highly resistant to pyrethroids but still susceptible to organophosphate. The usage of synergists such as piperonyl butoxide (PBO) could be considered in order to overcome the resistance due to oxidases. Proactive monitoring of the *kdr* gene throughout all dengue-endemic area in Malaysia is highly suggested as well.

## Figures and Tables

**Table 1:** The collection sites of the field-collected mosquitoes.

Name of Site	Assigned Name
Taman Seri Bayu	TSB
Ridzuan Condominium	RC
Flat Camar	FC
Taman Dahlia	TD

**Table 2:** The susceptibility of the permethrin-selected (IMR-PSS) *Aedes aegypti* (F1–F7) third instar larvae to permethrin. LC: Lethal concentration; CL: Confidence Limit.

Filial Generation	LC_50_ (mg/L)	CL* (mg/L) (Min,Max)	LC_90_ (mg/L)	CL* (mg/L) (Min,Max)	Regression
IMR-PSS F0	0.148	0.13,0.17	0.37	0.30,0.43	Y = 5.834X − 4.844
IMR-PSS F1	0.146	0.19,0.17	0.42	0.33,0.58	Y = 5.123X − 4.284
IMR-PSS F2	0.180	0.16,0.21	0.62	0.48,0.89	Y = 4.336X − 3.233
IMR-PSS F3	0.202	0.16,0.26	7.87	3.93,23.02	Y = 1.461X + 1.016
IMR-PSS F4	0.435	0.50,0.38	1.31	0.97,2.24	Y = 4.862X + 1.756
IMR-PSS F5	0.660	0.58,0.77	1.83	1.38,2.99	Y = 5.24X + 0.947
IMR-PSS F6	0.930	0.54,2.23	51.73	12.12,102.56	Y = 1.33X + 0.42
IMR-PSS F7	1.020	0.70,1.80	18.53	7.10,109.40	Y = 1.847X − 0.016

**Table 3:** The permethrin susceptibility of the laboratory-susceptible (IMR-LS) *Aedes aegypti* (F1061–F1066) third instar larvae to permethrin. LC: Lethal concentration; CL: Confidence Limit.

Filial Generation	LC_50_ (mg/L)	CL* (mg/L) (Min,Max)	LC_90_ (mg/L)	CL* (mg/L) (Min,Max)	Regression
IMR-LS F1061	0.012	0.010,0.015	0.029	0.021,0.059	Y = 2.618X + 11.62
IMR-LS F1062	0.010	0.009,0.012	0.018	0.015,0.027	Y = 3.969X + 18.32
IMR-LS F1063	0.010	0.009,0.011	0.018	0.015,0.027	Y = 3.986X + 18.32
IMR-LS F1064	0.010	0.009,0.012	0.018	0.015,0.027	Y = 3.969X + 18.33
IMR-LS F1065	0.011	0.009,0.021	0.023	0.015,0.0.31	Y = 3.078X + 13.88
IMR-LS F1066	0.012	0.010,0.016	0.0032	0.022,0.085	Y = 2.472X + 10.83

**Table 4:** The susceptibility of IMR-LS (generation: F1066) and IMR-PSS (generation: F7) to pyrethroids.

Mosquito Strain	Insecticide	KT_50_ (Min)	Regression	CL (Min,Max)
IMR-LS F1066	Permethrin	24.30	Y = 7.642X − 10.593	22.76,25.84
	Deltamethrin	12.08	Y = 8.523X − 9.222	10.92,13.15
	Cyfluthrin	10.62	Y = 8.736X − 8.964	14.77,17.27
	Lambda-cyhalothrin	16.63	Y = 8.91X − 10.85	14.78,17.25
IMR-PSS F7	Permethrin	ND	ND	ND
	Deltamethrin	56.95	Y = 3.99X − 7.02	53.03,62.46
	Cyfluthrin	43.17	Y = 3.96X − 6.53	42.78,48.04
	Lambda-cyhalothrin	87.73	Y = 3.758X − 7.303	74.75,114.60

KT: Knockdown time; CL: Confidence Limit; NA: Not Determined by Probit analysis due to the low knockdown rate (<5%), indicating a high resistance against permethrin.

**Table 5:** The overall classification of the enzyme activity in IMR-PSS and IMR-LS strains of adult *Aedes aegypti*.

Enzyme Activity	Strain	High (+++)	Moderate (++)	Low (+)
MFO assay	IMR-PSS	54%	30%	16%
	IMR-LS	0%	0%	100%
	TD	0%	21%	79%
	FC	66%	34%	0%
	TSB	0%	33%	67%
Non-specific esterase assay	IMR-PSS	4%	0%	96%
	IMR-LS	0%	0%	100%
AchE assay	IMR-PSS	17%	27%	56%
	IMR-LS	9%	14%	77%

MFO activity is expressed in nmol cytochrome C/min/mg of protein mg, high: >0.7, moderate: 0.4–0.7, low: 0.1–0.3; Non-specific esterase activity is expressed in nmol α-naphthol/min/mg of protein, high: >70, moderate: 20–69, low: <20; AChE assay is expressed in % of Propoxur inhibited fraction, high: >81%, moderate: 61–80, low: <60%.
